# Development of the Japanese Version of the Self-Endangering Work Behavior (J-SEWB) Scale

**DOI:** 10.14789/jmj.JMJ21-0039-OA

**Published:** 2022-05-27

**Authors:** KAZUHITO YOKOYAMA, AKINORI NAKATA, YUTO KANNARI, FRANK NICKEL, NICOLE DECI, ANDREAS KRAUSE, JAN DETTMERS

**Affiliations:** 1 Department of Epidemiology and Social Medicine, Graduate School of Public Health, International University of Health and Welfare, Tokyo, Japan; 1 Department of Epidemiology and Social Medicine, Graduate School of Public Health, International University of Health and Welfare, Tokyo, Japan; 2 Department of Epidemiology and Environmental Health, Juntendo University Faculty of Medicine, Tokyo, Japan; 2 Department of Epidemiology and Environmental Health, Juntendo University Faculty of Medicine, Tokyo, Japan; 3 Department of Social Medicine, Graduate School of Medicine, International University of Health and Welfare, Tokyo, Japan; 3 Department of Social Medicine, Graduate School of Medicine, International University of Health and Welfare, Tokyo, Japan; 4 Utsunomiya Campus Liberal Arts Center, Teikyo University, Tochigi, Japan; 4 Utsunomiya Campus Liberal Arts Center, Teikyo University, Tochigi, Japan; 5 Department of Foreign Languages, Institute of Foreign Languages, Teikyo University, Tokyo, Japan; 5 Department of Foreign Languages, Institute of Foreign Languages, Teikyo University, Tokyo, Japan; 6 MSH Medical School Hamburg, University of Applied Sciences and Medical University, Department of Psychology, Hamburg, Germany; 6 MSH Medical School Hamburg, University of Applied Sciences and Medical University, Department of Psychology, Hamburg, Germany; 7 School of Applied Psychology, University of Applied Sciences and Arts Northwestern Switzerland, Olten, Switzerland; 7 School of Applied Psychology, University of Applied Sciences and Arts Northwestern Switzerland, Olten, Switzerland; 8 Faculty for Psychology, University of Hagen, Germany; 8 Faculty for Psychology, University of Hagen, Germany

**Keywords:** self-endangering work behavior, overwork, presenteeism, flexible work style

## Abstract

**Objective:**

The concept of self-endangering work behavior (SEWB) was recently proposed to describe problematic behaviors to cope with heavy workloads and self-management. Although SEWB may enable workers to achieve immediate goals, it risks health and long-term work capacity. In this study, we developed a Japanese version of the SEWB (J-SEWB) scale, which was originally in German, and verified its validity and reliability.

**Materials:**

The original SEWB scale consisted of 21 items, constituting five subscales: “Intensification of working hours,” “Prolongation/extension of working hours,” “Refraining from recovery/leisure activities,” “Working despite illness,” and “Use of stimulating substances.” We translated the scale into Japanese, then checked the wording using back-translation.

**Methods:**

The J-SEWB scale and questions for working conditions and sociodemographic variables was administered via an online survey with 600 participants registered with an internet survey company in Japan. Cronbach's α coefficients were calculated for each subscale to assess internal consistency. Construct validity was examined using principal factor analysis with equamax rotation. An analysis of variance evaluated the relationships of J-SEWB scores with working conditions and sociodemographic variables.

**Results:**

Cronbach's α coefficients ranged from 0.846 to 0.964 for five subscales, and 0.957 for all 21 items (total J-SEWB score) in 600 participants. The factor analysis identified five factors, classifying 21 items into corresponding subscales. Total J-SEWB scores were significantly higher for flexible work as well as longer working hours.

**Conclusions:**

The J-SEWB scale appears to be an effective tool for assessing SEWB in Japanese employees, with satisfactory reliability and construct validity

## Introduction

In Japan, the needs of workers are diversifying^1)^ and the government is promoting flexible work styles, such as telework, side jobs and freelance work, establishing new work styles and promoting regional revitalization through remote work^2)^. The proportion of remote workers is increasing, and remote work is estimated to account for more than 20% of the working population in Japan^3)^. The demand for such flexible working styles is increasing worldwide, and companies are giving workers more control over their daily work based on self-management and self-discipline^4, 5)^. While these changes provide opportunities for personal growth and coordination between work and personal life, they also require the need for self-management, and can lead to problematic behaviors to cope with heavy workloads^6-8)^. This coping behavior is referred to as self-endangering work behavior (SEWB), which refers to work behaviors that may help achieve immediate goals while simultaneously creating risks for employee health and long-term work capacity^6-8)^.

The concept of SEWB is a combination of several maladaptive coping styles that have so far been studied separately, such as extension of working hours, intensification of working hours, sickness presenteeism, faking, substance abuse to recuperate, substance abuse to perform, reduction of quality, and bypassing safety standards^6-8)^ A central predictor for the occurrence of SEWB is increased results-oriented management in companies (indirect control instead of command-and-control)^9, 10)^. In workplaces using this type of management, employees are given freedom to make decisions, but at the same time they are responsible for achieving demanding goals. Knecht et al.^11)^ reported that SEWB contributed to the link between work burden and exhaustion in 607 workers under indirect control. Baeriswyl et al.^12)^ confirmed these results in a sample of 560 teachers, reporting that extension of working hours partially mediated the effect of workload on emotional exhaustion. Steidelmüller et al.^13)^ reported that the frequency of presenteeism (working despite illness) increased with the number of hours of teleworking per week among 25,465 respondents in the 6th wave of the European Working Conditions Survey 2015.

To quantitatively evaluate the SEWB, some of the authors of the present study^6, 8)^ created a self-administered questionnaire called the SEWB scale, in German, consisting of 21 items with a 5-step Likert scale, which included five subscales: “Intensification of working hours,” “Prolongation/extension of working hours,” “Refraining from recovery/leisure activities,” “Working despite illness,” and “Use of stimulating substances.” Using this scale, the authors found that emotional exhaustion and psychosomatic complaints of workers were increased by SEWB (excluding “Extension of working hours”) in 485 professionals, including engineers, architects, computer engineers, advertisers, and lawyers as well as scientists. The study showed the relationship between stressors and exhaustion is partially mediated by SEWB. Another study^14)^ reported that SEWB may moderate the relationship between alleged challenge stressors such as time pressure, irritation and work engagement, increasing the strain effect and reducing the challenge effect of time pressure.

In the current study, we developed a Japanese version of the SEWB scale (J-SEWB) to enable further research in Japanese employees regarding work behavior and health status, as the population of employees engaging in flexible work is rapidly increasing. Understanding the processes by which flexible work arrangements can lead to health impairments via maladaptive coping behaviors such as SEWB may help to better achieve the benefits of flexible work without the negative health impacts in Japan.

## Materials and Methods

### Self-Endangering Work Behavior (SEWB) scale

The SEWB scale was developed as a self-administered questionnaire consisting of 21 items, including five subscales: “Intensification of working hours,” “Prolongation/extension of working hours,” “Refraining from recovery/leisure activities,” “Working despite illness,” and “Use of stimulating substances.” The five subscales contain 3, 4, 6, 5, and 3 items, respectively. All of the self-endangering items were scored on a five-point Likert scale that ranged from 1 (rarely/never) to 5 (very often). Respondents were asked to report the frequencies of various behaviors, such as working despite illness. The scores of the items were totaled and used as the subscale score. The total score of the five subscale scores was taken as the total SEWB score. The process of selecting questionnaire items and validation as well as English translation of 21 items was reported previously by some of the authors of the present study^6, 8)^.

### Translation

First, the German version of the SEWB scale was translated into Japanese by one of the authors (YK). Next, a bilingual (German and Japanese) author (FN), who had not read the original items, conducted back-translation into German. The German author of this study (JD) examined the quality of translated versions, having compared them with the original German version. Based on this author’s evaluation, corrections were made for words, meanings, and item content by the authors (KY, YK, and FN) with the assistance of a Japanese employee in JD’s laboratory. The final items of the J-SEWB scale are listed in the Appendix.

### Survey questionnaire and protocol

The questionnaire used in the current study consisted of the J-SEWB scale and questions regarding sociodemographic variables, such as age, gender, job, work conditions, and annual income. The internet survey was outsourced to a research company (hamon Inc, Yokohama, Japan). Of the 1,052,566 registered individuals, 4,057 full-time employees aged 20 to 64 years who worked 30 hours or more a week were randomly selected (2,399 men and 1,658 women). These respondents were asked to answer an online questionnaire from September 8th, 2021, adjusting the number of responses by age group to be similar to the result of Labor Force Survey in Japan (2020)^15)^. The survey was discontinued when the total number of answers reached 600; this sample size was the maximum that the research budget allowed, and exceeded the size (300 or more) that would give stable results in factor analysis^16)^. Other than listed here, there were no conditions to include or exclude study subjects.

### Statistical analysis

The Cronbach’s α coefficient was calculated for each subscale to assess the internal consistency. Construct validity was examined using principal factor analysis with equamax rotation. Relationships among J-SEWB scores and sociodemographic variables were examined using t-test, χ^2^ test, or analysis of variance. All of the statistical analyses were conducted using IBM SPSS version 26.0.

### Ethical issue

This study was conducted after approval by the International University of Health and Welfare Research Ethics Committee (21-Ig-13, May 19, 2021). Participants agreed to participate voluntarily in the survey under a contract with the research company and were anonymous to us; we did not get informed consent from each.

## Results

Sociodemographic characteristics of 600 participants by gender are shown in Table 1. The proportions of participants in each age group were 18.3%, 21.2%, 27.3%, 24.1%, and 9% for the 20–29, 30–39, 40–49, 50–59 and 60–64 years age groups, respectively. Participants were mainly office workers (76.2%) and public officials (10.8%) with a smaller proportion of professionals and faculty/researchers (5.7%). Working hours per week were 30–49 hours for most of participants (86.3%), whereas a small proportion (13.7%) reported working 50 hours or more per week. Approximately 30% of participants worked under a non-fixed (flexible) working hours system, whereas the remaining 70% worked under a fixed working hours system. Half of the participants earned 4 million Yen or more per year.

**Table 1:** Sociodemographic characteristics of 600 participants (329 men and 271 women)

	Men	Women	Total
Age (years):			
20–29	57	53	110
30–39	70	57	127
40–49	91	73	164
50–59	79	66	145
60–64	32	22	54
Job:			
Public official (office work)	23	23	46
Public official (technical)	18	1	19
Office worker (office work)	79	140	219
Office worker (technical)	78	20	98
Office worker (sales)	25	12	37
Office worker (other)	65	38	103
Professionals (e.g., doctors, lawyers)	12	16	28
Faculty / Researcher	2	4	6
Working hours/week:			
30–39	83	122	205
40–49	180	133	313
50–59	49	13	62
60 or more	17	3	20
Working hour system:			
Fixed	212	197	409
Non-fixed:			
Variable	30	32	62
Flextime	57	28	85
Exemption	22	9	31
Advanced professional type	2	0	2
Others	6	5	11
Annual income in 2020 (1,000 Yen):			
less than 2,000	13	36	49
2,000–3,999	95	156	251
4,000–7,999	171	73	244
8,000–11,999	39	5	44
12,000 or more	11	1	12

Table 2 shows scores and Cronbach’s α coefficients for the J-SEWB scale. Cronbach’s α coefficients were 0.846 to 0.946 for five subscales, and were 0.958 (men), 0.951 (women), and 0.957 (combined) for total SEWB scores. Average scores on the total SEWB and five subscales in men were higher than those in women. These differences were statistically significant (t-test, p < 0.05) except for “Intensification of working hours” and “Use of stimulating substances” (p > 0.05).

**Table 2:** Scores and Cronbach's alpha coefficients of the J-SEWB scale in 600 participants

	Average	SD	Min	Max	Cronbach's alpha
Men:					
Intensification of working hours	7.2	2.8	3	15	0.891
Prolongation/extension of working hours	8.7	3.7	4	20	0.848
Refraining from recovery/leisure activities	12.3	5.2	6	30	0.934
Working despite illness	9.3	4.9	5	25	0.953
Use of stimulating substances	6.5	3.1	3	15	0.955
Total SEWB	43.9	16.3	21	105	0.958
					
Women:					
Intensification of working hours	6.8	3.1	3	15	0.933
Prolongation/extension of working hours	7.3	3.5	4	20	0.834
Refraining from recovery/leisure activities	9.9	4.6	6	27	0.933
Working despite illness	8.0	4.1	5	25	0.939
Use of stimulating substances	6.1	3.4	3	15	0.972
Total SEWB	38.0	15.0	21	92	0.951
					
Total:					
Intensification of working hours	7.0	2.9	3	15	0.912
Prolongation/extension of working hours	8.0	3.7	4	20	0.846
Refraining from recovery/leisure activities	11.2	5.1	6	30	0.937
Working despite illness	8.7	4.6	5	25	0.949
Use of stimulating substances	6.3	3.2	3	15	0.964
Total SEWB	41.2	16.0	21	105	0.957

**Figure 1:**
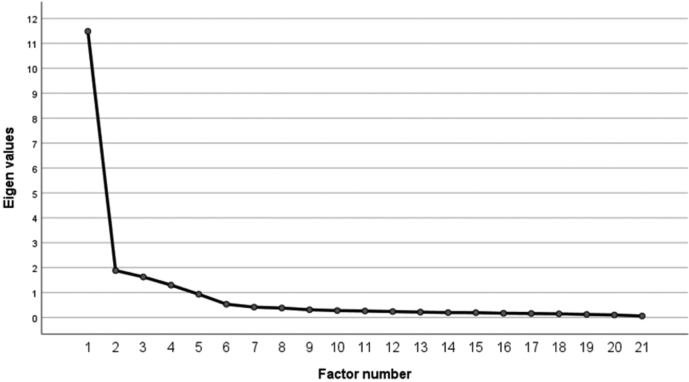
Scree plot of principal factor analysis on 21 items of the J-SEWB in 600 participants (before rotation)

Table 3 shows factor loadings for 21 items. A scree plot is drawn in the Figure 1. Five factors were extracted by factor analysis, classifying 21 items into their corresponding subscales, except that item 7, “I work more than 10 hours a day without being directed to do so,” which was most heavily loaded on “Prolongation/extension of working hours” in the original German scale, exhibited the highest loading on Factor 2 (“Refraining from recovery/leisure activities”).

**Table 3:** Factor loadings on 21 items of the J-SEWB (principal factor analysis with equamax rotation)

Subscales	Items	Factor 1	Factor 2	Factor 3	Factor 4	Factor 5
IW	1	.089	.123	.157	**.790**	.187
	2	.156	.183	.198	**.825**	.212
	3	.272	.201	.194	**.795**	.202
PW	4	.205	.155	.167	.266	**.733**
	5	.168	.204	.133	.171	**.848**
	6	.203	.455	.195	.255	**.514**
	7	.160	**.523**	.184	.369	.306
RR	8	.260	**.563**	.229	.240	.441
	9	.225	**.576**	.151	.384	.230
	10	.287	**.682**	.214	.273	.325
	11	.323	**.657**	.211	.251	.333
	12	.354	**.699**	.217	.191	.314
	13	.370	**.656**	.217	.196	.353
WI	14	**.715**	.250	.199	.260	.243
	15	**.676**	.339	.252	.161	.277
	16	**.747**	.337	.227	.158	.206
	17	**.833**	.214	.210	.217	.237
	18	**.811**	.164	.209	.255	.249
US	19	.191	.142	**.853**	.173	.171
	20	.147	.115	**.927**	.153	.129
	21	.135	.119	**.926**	.165	.113

Subscales: IW = Intensification of working hours PW = Prolongation/extension of working hours RR = Refraining from recovery/leisure activities WI = Working despite illness US = Use of stimulating substances

Table 4 shows the sociodemographic variables that were significantly related to the total J-SEWB scores in a two-way analysis of variance. The total J-SEWB scores were significantly higher for flexible work and longer working hours. The proportion of participants working with flexible hours significantly increased as working hours prolonged.

**Table 4:** Sociodemographic variables significantly related to J-SEWB scores in 600 participants: Two-way analysis of variance

	J-SEWB scores		F-values^a^	
	Mean	SD	Variable	Gender
Working hour system:			11.55*	18.88**
Fixed	39.6	16.0		
Non-fixed	44.8	15.6		
Working hours/week^b^:			6.94**	13.20**
30–39	39.2	16.0		
40–49	40.6	15.4		
50–59	46.6	15.3		
60 or more	56.0	17.9		

*p < 0.01, **p < 0.001 ^a^ Two-way analysis of variance using gender and each variable as factors (main effects). ^b^ Among participants who worked 40 hours or less, 40–49 hours, 50–59 hours, and 60 or more hours a week, 56 (27.3%), 98 (31.3%), 28 (45.2%), and 9 (45.0%) were working under a flexible working hour system (χ^2^ = 8.640, p < 0.05).

## Discussion

The J-SEWB scale showed good reliability, as indicated by Cronbach’s α coefficients of 0.846 or above for all five subscales and the total scores. The factor analysis identified five factors, classifying 21 items into their corresponding subscales with one exception, indicating that the construct validity was satisfactory. Furthermore, the total J-SEWB scores were significantly higher with flexible work and longer working hours; the proportion of participants working with flexible hours significantly increased as working hours prolonged. This suggests that the J-SEWB scale reflected overwork, possibly related to flexible work conditions. Thus, the J-SEWB scale appears to be an effective tool for assessing SEWB related to autonomy and self-management in Japanese employees. It should be emphasized that these results were obtained from subjects with the same age structure as the labor force in whole Japan^15)^.

Among four items of “Prolongation/extension of working hours,” item 7, “I work more than 10 hours a day without being directed to do so,” was most heavily loaded on “Refraining from recovery/leisure activities” in the present study. A positive response to item 7 indicates that employees work for a long time, while the other three items (No. 4–6) indicate a tendency to work even in leisure time. The results for item 7 may indicate that work interferes with leisure time and causes recreation to be abandoned.

The total SEWB and subscale scores in men were significantly higher than those in women, except for the two subscales, “Intensification of working hours” and “Use of stimulating substances.” The gender differences in the total SEWB scores were still significant after controlling for the effects of working hours as well as the working hours system (fixed or non-fixed) in a two-way analysis of variance. Although the reasons for this gender difference were not investigated in the current study, this finding could potentially be related to the working environment of female workers in Japanese companies. In Japan, the proportion of managerial positions occupied by women was only 14.8% in 2019, which is much lower than that in many other countries^17)^. The lower degree of flexibility at work might have been underlying the lower SEWB scores in women in the present study.

SEWB is an active coping behavior that has adverse health effects for workers, although it is conventionally believed that active coping behaviors mitigate the psychosomatic effects of stress in contrast to avoidance coping behaviors^7, 8)^. SEWB might resemble psychological states such as work engagement and overcommitment, in terms of overwork. However, SEWB is a specific observable behavior that may mediate the effects of overload on health impairment^7, 8)^. Thus, in addition to the occupational stressors and psychosocial modifiers related to the health effects on employees, which have been extensively studied in Japan^18-21)^, it may be valuable for future studies to examine SEWB as a coping strategy for flexible working styles. Such studies will also verify the concurrent validity of J-SEWB scale by examining its relationships to the existing scales^18-21)^, which could not be done due to insufficient preparation in the present study.

The increased incidence of SEWB in the workplace may indicate that the design of flexible forms of work needs to be improved. In such cases, determinants of SEWB in relation to occupational stressors and modifiers, including gender, should be investigated to reveal how to improve them. The J-SEWB scale can be used to assess determinants as well as the effectiveness of interventions and may be helpful in health management of workers. A previous study proposed that long working hours may have a cultural background that is specific to Japan, such as an emphasis on signals that show commitment/loyalty to the company and one’s efforts for others, rather than results/achievements, groupism, hierarchical relationships, and workload unrelated to core business^22)^. An international comparative study on the SEWB that considers differences in workplace culture in future may provide valuable insight.

## Conclusions

The J-SEWB scale appears to be an effective tool for assessing SEWB related to autonomy and self-management in Japanese employees, with satisfactory reliability and construct validity.

## Funding

This study was supported by JSPS KAKENHI Grant number 22K10534, 19H01763 and 20K20869.

## Author contributions

KY contributed to all aspects of the study, including research design, data collection and analysis, and writing the manuscript. JD and AN designed the study with KY.

JD, ND, AK provided the original German version of SEWB scale. The translation was performed by YK, FN, JD, and KY. All of the authors participated in writing the manuscript.

## Conflicts of interest statement

The authors declare that there are no conflicts of interest.

## Supplementary Material

Appendix Subscales and items of the J-SEWB scale
