# Supplementary figures and images for: Development of the Japanese Version of the Self-Endangering Work Behavior (J-SEWB) Scale
Source: Juntendo Iji Zasshi. 2022 May 27;68(3):242–50. doi: 10.14789/jmj.JMJ21-0039-OA (PMC11250010; doi:10.14789/jmj.JMJ21-0039-OA)

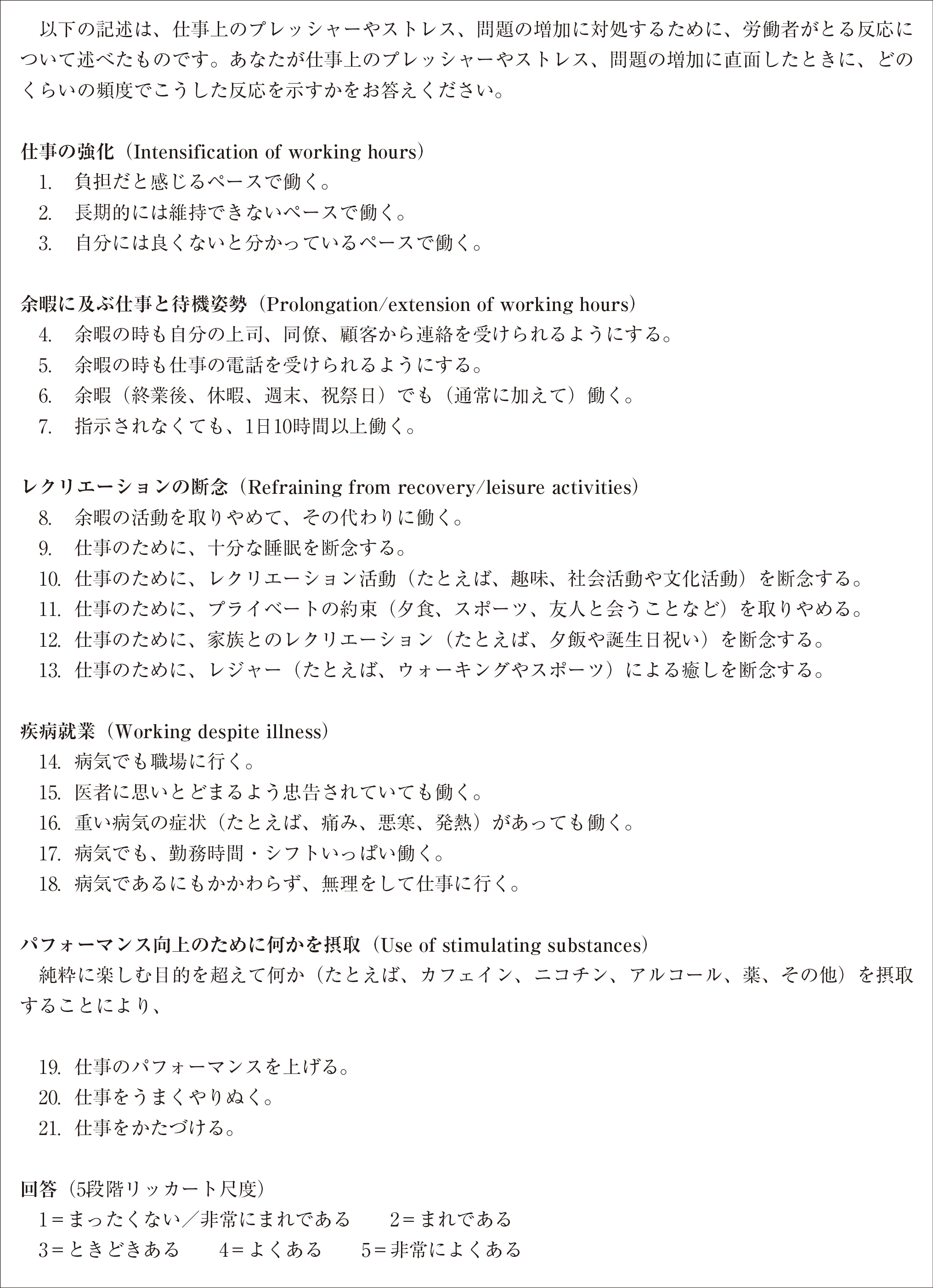

Supplement: Appendix — Subscales and items of the J-SEWB scale [file 2188-2126-68-3-0242-s001.jpg]
